# The Chinese Adaptation of the Teachers’ Sense of Efficacy Scale in Early Childhood Pre-Service Teachers: Validity, Measurement Invariance, and Reliability

**DOI:** 10.3390/bs15030329

**Published:** 2025-03-07

**Authors:** Mingxing Shao, Mohd Mokhtar Muhamad, Fazilah Razali, Nasnoor Juzaily Mohd Nasiruddin, Xinchong Sha, Guoqiang Yin

**Affiliations:** 1 Faculty of Educational Studies, Universiti Putra Malaysia, Serdang 43400, Malaysia; shaomingxing@mail.qtnu.edu.cn (M.S.); mk_mokhtar@upm.edu.my (M.M.M.); fazilahrazali@upm.edu.my (F.R.); 2 School of Preschool Education, Qiongtai Normal University, Haikou 571127, China; 3 Faculty of Sports and Exercise Science, University of Malaya, Kuala Lumpur 50603, Malaysia; nasnoorjuzaily@um.edu.my; 4 School of Education, Central China Normal University, Wuhan 430079, China

**Keywords:** teachers’ sense of efficacy, teachers’ sense of efficacy scale, early childhood pre-service teachers, psychometric properties

## Abstract

Teachers’ sense of efficacy (TSE) is a crucial construct for evaluating the quality of pre-service teachers. While the Teachers’ Sense of Efficacy Scale (TSES) is the most widely used and promising instrument for measuring TSE, there is no existing literature assessing the appropriateness of the TSES for early childhood pre-service teachers in China. This study aimed to adapt the English version of the TSES for the Chinese early childhood education contexts, testing its factor structure, validity, measurement invariance, and reliability. The sample included 402 participants in China. The TSES was translated into Chinese using the standard back-to-back translation method. CFA results indicated that the TSES is best represented by a modified three-factor model, demonstrating strong preliminary, overall, and internal structure fit. The concurrent validity, convergent validity, criterion-related validity, internal consistency reliability, and composite reliability of the Chinese version of the TSES were robust. The measurement invariance across age and college year was also confirmed. This study addresses a gap in the literature by providing robust empirical evidence on the factor structure, validity, measurement invariance, and reliability of the Chinese version of the TSES for early childhood pre-service teachers, thereby enhancing understanding of TSE in Chinese-speaking Confucian culture and in early childhood education contexts.

## 1. Introduction

According to Bandura (1997), teachers’ sense of efficacy (TSE) is commonly considered a form of self-efficacy. For pre-service teachers (PSTs), TSE refers to their confidence in their capacity to organize and carry out teaching-related behaviors effectively (Tschannen-Moran & Hoy, 2001; Plourde, 2002). In recent years, there has been a growing body of research focused on the TSE of PSTs (Burgueño et al., 2019; Ma et al., 2019; Khairani & Makara, 2020; Abraham et al., 2021; Cetin-Dindar, 2022; Yim, 2023; Lim, 2023; Çiftçi & Topçu, 2023; Ding & Hong, 2023). Evidence suggests that PSTs need to cultivate a strong sense of efficacy as it is critical to their effective teaching practice and pedagogical knowledge (Woolfolk et al., 1990; Fives et al., 2007; Kim & Cho, 2014; Ding & Hong, 2023). Previous research has demonstrated that PSTs with greater self-efficacy tend to implement more effective pedagogical approaches (Joo et al., 2000; Kim et al., 2013; Lim, 2023). Additionally, evidence in the literature shows that teachers with greater self-efficacy also have a higher passion for teaching (Sak, 2015; Pereira & Tay, 2023).

High-quality teachers are the cornerstone of education. The quality of early childhood PSTs (EC-PSTs) has gained renewed focus with the introduction of key education policies in China, such as the Professional Competency Standards of Normal Students of Preschool Education (2021), the Opinions on the Implementation of the Plan of Action for the Expansion and Quality of Basic Education in the New Era (2023), and the Law on Preschool Education (2024). These policies emphasize the importance of developing professional competencies in EC-PSTs to meet the evolving demands of early childhood education (ECE). TSE has been recognized as a crucial construct for evaluating PSTs’ quality, serving as an assessment of their capacity to implement effective instructional practices (Klassen et al., 2009; Pintus et al., 2021). For EC-PSTs specifically, TSE has been identified as a very strong indicator of their future effective instructional practices (Lin & Gorrell, 2001; Sak, 2015). Thus, it is vital to study EC-PSTs’ TSE to support the development of effective and high-quality early childhood teaching professionals in China.

### 1.1. Measurement of TSE

Over the past few decades, studies about the conceptualization and measurement structure of TSE have been mainly based on Bandura’s social cognitive theory and the construct of self-efficacy. According to this theory, self-efficacy is conceptualized as two different expectations: self-efficacy expectancy (i.e., “organize and execute the courses of action required to produce given attainments” (Bandura, 1997, p. 3) and outcome expectancy (i.e., “individual’s estimate of the likely consequences of performing that task at the expected level of competence” (Tschannen-Moran & Hoy, 2001, p. 787). Subsequently, various instruments have been developed to assess TSE according to this framework, such as the Teacher Efficacy Scale (Gibson & Dembo, 1984), Bandura’s Teacher Self-Efficacy Scale (Bandura, 1997), and the Science Teaching Efficacy Belief Instrument B (Enochs & Riggs, 1990). However, the construct validity and reliability of these instruments, as well as their meaning and utility, have been called into question by subsequent research (Tschannen-Moran et al., 1998, Tschannen-Moran & Hoy, 2001). In response to the challenge of how best to conceptualize and measure TSE, Tschannen-Moran et al. (1998); Tschannen-Moran and Hoy (2001) extended the concept of TSE and proposed an integrated conceptual model of TSE that incorporates Bandura’s self-efficacy and outcome expectancy, making the TSE more comprehensive by covering all facets of a teacher’s professional role. This model argues that TSE arises from the combined influence of evaluating “the analysis of the teaching task” and reflecting on one’s “self-perception of teaching competence” (Tschannen-Moran et al., 1998, p. 228). They then developed the Teachers’ Sense of Efficacy Scale (TSES) (Tschannen-Moran & Hoy, 2001) based on this model in American contexts (originally called the Ohio State Teacher Efficacy Scale). The TSES, which includes a 12-item short form and a 24-item long form, is the most widely used and promising instrument for measuring TSE (A. W. Hoy & Spero, 2005; Poulou, 2007; Knoublauch & Hoy, 2008; Klassen et al., 2009; Tsigilis et al., 2010; Yilmaz, 2011; Klassen et al., 2011; Duffin et al., 2012; Valls et al., 2020; Pintus et al., 2021; Salas-Rodríguez et al., 2021) as it aligns with Bandura’s theory and the recommendations of critics (Fives & Buehl, 2010; Klassen et al., 2011; Ma et al., 2019). According to Duffin et al. (2012), the TSES has become the predominant instrument for evaluating PSTs’ TSE.

### 1.2. Cross-Cultural Factor Structure Issues

A review of previous studies indicates that while both forms of TSES exhibit excellent internal consistency reliability among in-service teachers (ISTs) and PSTs (Tschannen-Moran & Hoy, 2001; Tsigilis et al., 2010; Salas-Rodríguez et al., 2021; Karami et al., 2021), the short form shows better psychometric properties and cross-cultural adaptation (Ruan et al., 2015; Salas-Rodríguez et al., 2021; Monteiro & Forlin, 2023). However, there is significant debate and conflicting statistical outcomes regarding the factor structure of short-form TSES (TSES-SF) when utilized with PSTs from various countries (Tschannen-Moran & Hoy, 2001; Poulou, 2007; Fives & Buehl, 2010; Burgueño et al., 2019; Chan et al., 2024), indicating that the factor structure of the scale remains unresolved.

Prior studies have extensively examined the TSES-SF’s factor structure across various cultural contexts. Besides the initial three-factor model—“Efficacy for instructional strategies (IS)”, “Efficacy for classroom management (CM)”, and “Efficacy for student engagement (SE)”—proposed by Tschannen-Moran and Hoy (2001) in American contexts, researchers from different cultural settings have suggested alternative models, highlighting cross-cultural variations in the factor structure of the scale. For example, Tsui and Kennedy (2009) recommended a two-factor model based on Hong Kong ISTs. Ruan et al. (2015) proposed a modified three-factor model based on ISTs from China, Korea, and Japan by removing item 8. This model has also recently been found applicable in Spanish and Vietnamese contexts (Burgueño et al., 2019; V. T. Ho et al., 2023). Building on this model, Lu et al. (2021) introduced another edition of the three-factor model with 11 items based on Chinese special education ISTs by revising item 12 as an IS factor rather than a SE factor. Furthermore, Tschannen-Moran and Hoy (2001) noted that the single-factor model was more ideal for American PSTs than the three-factor model. This finding was validated by subsequent research that employed American PSTs as the sample (Fives & Buehl, 2010; Duffin et al., 2012). However, Burgueño et al. (2019) suggested that, for Spanish PSTs, the modified three-factor model recommended by Ruan et al. (2015) was more ideal. Recently, Chan et al. (2024) proposed a complex bi-factor model, suggesting it was preferred for Australian EC-PSTs.

Previous research has demonstrated that the construct of TSE is heavily influenced by cultural factors, such as the beliefs regarding the role of teachers (Lin & Gorrell, 2001) and differences in samples between PSTs and ISTs (Tsui & Kennedy, 2009). In the context of Confucianism, ISTs in Hong Kong exhibit a greater sense of moral responsibility in guiding students in their daily lives (I. T. Ho & Hau, 2004). They also tend to be more concerned with students’ academic performance, and they generally believe that discipline or classroom management is a prerequisite for effective teaching and learning (Tsui & Kennedy, 2009). In mainland China, ISTs’ classroom teaching, rooted in Confucian cultural traditions, places a strong emphasis on classroom discipline and is characterized by collectivism (Smith & Hu, 2013). The variations in factor structures across the studies mentioned highlight the importance of understanding TSE within specific cultural and educational contexts. Another significant gap in the literature is the lack of studies assessing the appropriateness of the TSES for EC-PSTs in China. While the TSES has been widely validated in various international contexts in prior studies, its cultural adaptation and applicability specifically within the contexts of ECE and EC-PSTs in China remain under-explored. The unique cultural and educational characteristics of the Chinese ECE contexts, rooted in Confucian cultural traditions, may influence how EC-PSTs perceive and demonstrate TSE. As the TSES-SF was originally developed adopting American samples, certain items or factor structures may not be suitable or applicable to EC-PSTs in the context of Chinese Confucian culture. Addressing this gap is essential for accurately assessing TSE and understanding the unique challenges faced by EC-PSTs in China’s rapidly evolving educational landscape.

### 1.3. The Current Research

Given that TSE is context-dependent and may vary according to cultural values and demographic variables such as gender and teaching field (Dilekli & Tezci, 2020), this study aims to adapt the English version of the TSES for Chinese ECE contexts, testing its factor structure and psychometric properties, including construct validity, measurement invariance across age and college year, concurrent validity, convergent validity, and criterion-related validity (via its link to professional identity [PI]), as well as internal consistency reliability and composite reliability among Chinese EC-PSTs. This is essential for supporting and improving the quality of EC-PSTs in Chinese-speaking contexts.

According to Marschall (2022), PI plays an important role in supporting the development of PSTs’ TSE. Meanwhile, TSE can positively predict the PI of teachers (Zarrinabadi et al., 2023). Furthermore, in the field of ECE, TSE is also believed to be associated with the PI of teachers (Matsuda & Hamada, 2024). The above evidence offers some insight into the relationship between PI and TSE, which implies that PI may be a good criterion for testing TSE. In the present study, the PI was measured using the Student Teacher Professional Identity Scale (STPIS) due to the STPIS having been widely used to measure PI in PSTs, including EC-PSTs, in China (X. Q. Wang et al., 2017, 2018; L. Y. Zhang et al., 2018; Y. Wang et al., 2022). Moreover, the TSES-SF and STPIS were chosen as part of a larger quasi-experimental study on the effects of different instructional strategies on EC-PSTs’ TSE and PI in Hainan, China.

## 2. Materials and Methods

### 2.1. Research Design and Participants

This study employed a cross-sectional survey methodological design to assess the TSE of EC-PSTs at a normal university in Hainan province, China^1^. Data were collected from 402 ECE undergraduate PSTs (ages ranged from 18 to 24, *M_age_* = 20.42, *SD* = 1.35) using a simple random sampling method. All were enrolled in a four-year (8 semesters) pre-school education major program at this normal university. During the four years, PSTs are required to complete 51.5 credits of general education courses, 24.5 credits of professional education courses, and 64 credits of teacher education courses. In addition, they must also fulfill 19 credits of comprehensive practical courses, which include an observation-based practicum (lasts one week) from the second to sixth semesters, a one-semester teaching practicum in the seventh semester, and the completion of a graduation thesis. The observation-based practicum primarily involves observation to understand and learn about the duties and procedures of early childhood education and care in kindergartens, while the teaching practicum requires a comprehensive, hands-on experience in kindergartens, focusing on strengthening the practical capabilities required for early childhood education and care.

While the sample was drawn from a single university, participants in this study were from 26 different provinces across China (Table 1), reflecting considerable demographic diversity. Additionally, all teacher education programs in China must adhere to national curriculum standards^2^, certification standards^3^, and undergraduate education evaluations^4^, which ensure a degree of consistency across universities, even within different provinces. Therefore, while our sample is regional, the findings may provide valuable insights into the broader contexts of ECE in China. Participants were 92.3% female (*n* = 371) and 7.7% male (*n* = 31). According to the latest data from the Ministry of Education of China, there were 3,244,204 full-time early childhood ISTs (EC-ISTs) in China as of 2022, with 316, 6616 (97.61%) being female (Ministry of Education of the People’s Republic of China, 2024). Additionally, as of 2024, the sampled normal university had 1946 EC-PSTs enrolled, of which 1823 (93.68%) were female. Therefore, the gender distribution of our sample was representative of the EC-PSTs in Hainan and EC-ISTs in China. Regarding college year, 23.40% (*n* = 94) of the respondents were freshmen (first year), 29.60% (*n* = 119) were sophomores (second year), 42.50% (*n* = 171) were juniors (third year), and 4.50% (*n* = 18) were seniors (fourth year). Sample sizes considered appropriate for factor analysis are typically more than 10 times the total number of items (Singh et al., 2016), or at least 300 cases (Tabachnick & Fidell, 2013). Given that the TSES-SF has 12 items, the sample size of this study (*n* = 402) was satisfactory for meeting the minimum requirement. Table 1 shows the sociodemographic descriptions of the sample.

### 2.2. Measures

#### 2.2.1. Teachers’ Sense of Efficacy Scale Short Form, TSES-SF

The measurement of EC-PSTs’ TSE was conducted using the TSES-SF with 12 items (Tschannen-Moran & Hoy, 2001). As Table 2 shows, all 12 items were categorized into three sub-scales: IS, CM, and SE. The TSES-SF was measured using a Likert 9-point scale, where 1 = “Nothing”, 2 = “Negligible”, 3 = “Very little”, 4 = “A slight degree”, 5 = “Some degree”, 6 = “A fair amount”, 7 = “Quite a bit”, 8 = “Almost a great deal”, and 9 = “A great deal”. The overall TSES score is derived from an average of the three factors. The *α* for TSES-SF in the present study was 0.93.

The TSES-SF was translated adequately from English into Chinese using back-to-back translation to minimize translation-related validity and reliability issues. This method is often used to ensure coherence between the original scale and the translated version (Behling & Law, 2000). First, a native Chinese-speaking scholar specializing in ECE curriculum and instruction translated the TSES-SF into Chinese to ensure that the terminology used in the questionnaire was applicable to ECE. Considering the specific characteristics of ECE in China, where children aged 3 to 6 years attend kindergartens, we replaced “students/student” with “幼儿” (children/child) in items 2, 3, 6, 7, 8, 9, 10, 11, and 12, and “school” with “幼儿园” (kindergarten) in item 12. Additionally, recognizing that ECE does not have academic performance requirements and uses play-based pedagogies (Bull & Bautista, 2018) and activities, we replaced “schoolwork” with “教育教学活动” (educational and instructional activities) in items 9 and 11. Second, the Chinese version of the TSES-SF was re-translated to English by a native English scholar in the field of psychology to ensure the accuracy of the translation in the previous step. Third, the authors and professionals familiar with English translation and educational psychology compared the two versions of the TSES-SF item by item to ensure that the meaning and grammatical structure of all items were maintained in translation. Finally, a Chinese psychology scholar and two Chinese education investigators revised and improved the TSES-SF for all the differences found in the comparison of the two versions in the previous step and reached a consensus on each item. And then, a final Chinese version of the TSES-SF (C-TSES-SF) was generated. Appendix A.1 and Appendix A.2 shows both versions of the TSES-SF.

#### 2.2.2. Student Teacher Professional Identity Scale, STPIS

EC-PSTs’ PI was measured using the Chinese version of STPIS (X. Q. Wang et al., 2013), with 12 items (e.g., “I think PSTs are respected”) on a Likert 5-point scale where 1 = “Strongly disagree”, 2 = “Disagree”, 3 = “Neither agree nor disagree”, 4 = “Agree”, and 5 = “Strongly agree”. The 12 items were divided into four sub-scales: “Professional willingness (PW)”, “Professional values (PVa)”, “Professional efficacy (PE)”, and “Professional volition (PVo)”. Higher scores suggest a stronger sense of PI. The *α* for STPIS was 0.77 in the present study.

### 2.3. Procedures

Before data collection, this study obtained ethical approval from the ethical committee of Universiti Putra Malaysia (Project ID: JKEUPM-2023-323) as well as permission for carrying out the study from the sampled normal university. All data were collected in the second half of the 2022–2023 school year. Participants were briefed on the aim of this research, who should not participate in this study, that the information they provided and their identity would be kept confidential, and who they should contact if they had additional questions about their participation in this study. All participants then voluntarily signed respondent’s information sheets and informed consent forms and then completed the questionnaires anonymously. No remuneration or compensation was offered to participants in the present study. The questionnaires were conducted online through the wjx.cn platform. As the wjx.cn platform includes a missing values notification feature, respondents were unable to submit incomplete questionnaires. Therefore, there were no missing values in the collected data.

### 2.4. Data Analysis

All of the data in this research were analyzed using SPSS version 22 and Amos version 24. Preliminary analyses, including *SD* and *mean*, were conducted. *Skewness* and *kurtosis* of the TSES-SF were utilized to examine its normality. As EFA is typically used for scale development, while CFA is more suitable when there is strong theoretical or empirical evidence, scholars recommend that CFA can be applied directly without prior EFA when such a foundation exists (Hurley et al., 1997). Given the consistency of the TSES with Bandura’s social cognitive theory and the previous empirical research on the factor structure of the TSES-SF, we employed CFA to investigate the six competing models of the TSES-SF among EC-PSTs. These included a one-factor model (“Model 1”: items 1–12) (Tschannen-Moran & Hoy, 2001), four multi-factor correlation structure models (“Model 2”: Teaching and Support [TS], items 1–4, 9, 10, 12; CM, items 5–8, 11 (Tsui & Kennedy, 2009). “Model 3”: IS, items 1–4; CM, items 5–8; SE, items 9–12 (Tschannen-Moran & Hoy, 2001). “Model 4”: IS, items 1–4; CM, items 5–7; SE, items 9–12 (Ruan et al., 2015). “Model 5”: IS, items 1, 2, 3, 4, 12; CM, items 5, 6, 7; SE, items 9, 10, 11) (Lu et al., 2021), and one higher-order factor structure model (“Model 6”: IS, items 1–4; CM, items 5–8; SE, items 9–11; General, items 1–12) (Chan et al., 2024).

We run CFA with the same sample (*n* = 402) to test the fitness of the factor structures of the six models, respectively. Prior to CFA, KMO tests (>0.80) and Bartlett’s test were conducted to determine the appropriateness of the sample for factor analysis. CFA was then performed using maximum likelihood estimation to validate the factorial structure of the six models. The assessment of the model’s adequacy was conducted utilizing the three dimensions of preliminary fit, overall fit, and internal structure fit advocated by Bagozzi and Yi (1988). According to Bagozzi and Yi (1988), the criteria for preliminary fit include all error variances of indicators being positive and the *p*-values being significant; standard errors are not “very large”; factor loadings are between 0.5 and 0.95. The overall model fit indices include *χ*^2^/df < 5 (Cronk, 2004), CFI > 0.95, TLI (NNFI) ≥ 0.95 (L. Hu & Bentler, 1999), RMSEA < 0.08 (Brown, 2015), GFI > 0.90 (Kline, 2005), NFI > 0.90 (Bentler & Bonett, 1980), IFI > 0.90 (Bollen, 1989), and the lower the value for AIC and ECVI, the better model fit (Duffin et al., 2012). The internal structure model fit indices include high individual items (>0.50), AVE > 0.50 (Fornell & Larcker, 1981; Bagozzi & Yi, 1988), and CR > 0.70 (Fornell & Larcker, 1981).

Following the selection of the suitable model, the measurement invariance of the C-TSES-SF model across age and college year, including configural, metric, scalar, and residual invariance (Widaman & Reise, 1997), was further evaluated by multigroup CFAs. For measurement invariance across age and college year, the sample was separated into two groups, respectively: age group 1 (18–20 years, *n* = 229) and age group 2 (21–24 years, *n* = 173); college year group 1 (freshmen [first year] and sophomores [second year], *n* = 213), and college year group 2 (juniors [third year] and seniors [fourth year], *n* = 189). The criteria of measurement invariance of C-TSES-SF are ΔCFI ≤ 0.01 and ΔRMSEA ≤ 0.015 (Cheung & Rensvold, 2002; Chen, 2007).

Next, we assessed the concurrent validity among C-TSES-SF’s three sub-scales, the convergent validity of the C-TSES-SF, as well as the criterion-related validity with the STPIS using two-tailed Pearson correlation analyses. Correlation strengths (*r*) were classified as follows: extremely high, *r* > 0.70; large, 0.30 < *r* < 0.70; medium, 0.10 < *r* < 0.30; low, *r* < 0.10 (Cohen, 1988). The concurrent validity criterion was *r* < 0.8 and *p* < 0.05 (Brown, 2015). Criteria for convergent validity include significant *p*-values, and larger (smaller) *r*-values indicate larger (smaller) convergent validity (Fornell & Larcker, 1981). The criterion-related validity would be supported if the correlation between the STPIS (including its four sub-scales) and the C-TSES-SF (including its three sub-scales) is positive and substantial, as J. Zhang (2019) and Hong et al. (2021) verified that self-efficacy was significantly positively correlated with PI.

Lastly, the reliability of the C-TSES-SF and its sub-scales were calculated using Cronbach alpha’s coefficient and composite reliability values (*ρ*) (Lentillon-Kaestner et al., 2018) for all participants (*n* = 402). Internal consistency was classified as follows: *α* > 0.7, acceptable; 0.9 > *α* > 0.8, good; *α* > 0.9, excellent (Terwee et al., 2007). Regarding *ρ*, an acceptable range is 0.60 ≤ *ρ* ≥ 0.70, a satisfactory range is 0.70 ≤ *ρ* ≥ 0.90, and an excellent range is *ρ* ≥ 0.90 (Nunnally & Bernstein, 1994).

## 3. Results

### 3.1. Preliminary Analyses

The normality of the sample was examined before conducting CFA. Table 3 presents descriptive statistics for all 12 items of the scale. Both *skewness* and *kurtosis* were in the range of −1 to +1, indicating that the data distributions of the TSES-SF were normal (Curran et al., 1996; Martínez Ortega et al., 2017).

### 3.2. Construct Validity

#### 3.2.1. The Preliminary Model Fit

The KMO statistic and Bartlett’s sphericity test outcomes illustrated that the factor analysis was appropriate, as KMO = 0.94 and Bartlett test, *χ*^2^ (66) = 3131.39 (*p* < 0.001). According to the CFA outcomes (*n* = 402), the preliminary fit of the first five models was good, except for Model 6. All error variances from e1 to e11 or e12 among the first five models were positive and significant (*p* < 0.001). However, in Model 6, the error variance e10 was negative (−1.45), and the p-values of e6 and e10 were not significant (*p* > 0.05). The *t*-values of e1 to e11 or e12 in the six models ranged from 8.65 to 13.50 (Model 1: 12.50 to 13.50, Model 2: 11.78 to 13.28, Model 3: 10.60 to 12.56, Model 4: 8.65 to 12.42, Model 5: 10.60 to 12.56, Model 6: −0.48 to 13.25). Standard errors of all parameters among the first five models ranged from 0.04 to 0.10 (Model 1: 0.04 to 0.10, Model 2: 0.04 to 0.10, Model 3: 0.04 to 0.08, Model 4: 0.04 to 0.08, Model 5: 0.04 to 0.08), and were not “very large”. However, in Model 6, the standard error of W7 (16.07) was more than 16 times the largest standard error in the first five models (0.10 in Model 1 and Model 2) and was “very large”. The standardized factor loadings between the latent variables and their measurement pointers among the first five models varied between 0.64 and 0.87 (Model 1: 0.64 to 0.80, Model 2: 0.66 to 0.82, Model 3: 0.71 to 0.84, Model 4: 0.71 to 0.87, Model 5: 0.69 to 0.87), meeting the criteria of greater than 0.50 and less than 0.95 (Bagozzi & Yi, 1988). However, in Model 6, the standardized factor loadings varied between 0.08 and 1.24, which did not meet the above criteria.

#### 3.2.2. The Overall Model Fit

**Figure 1:**
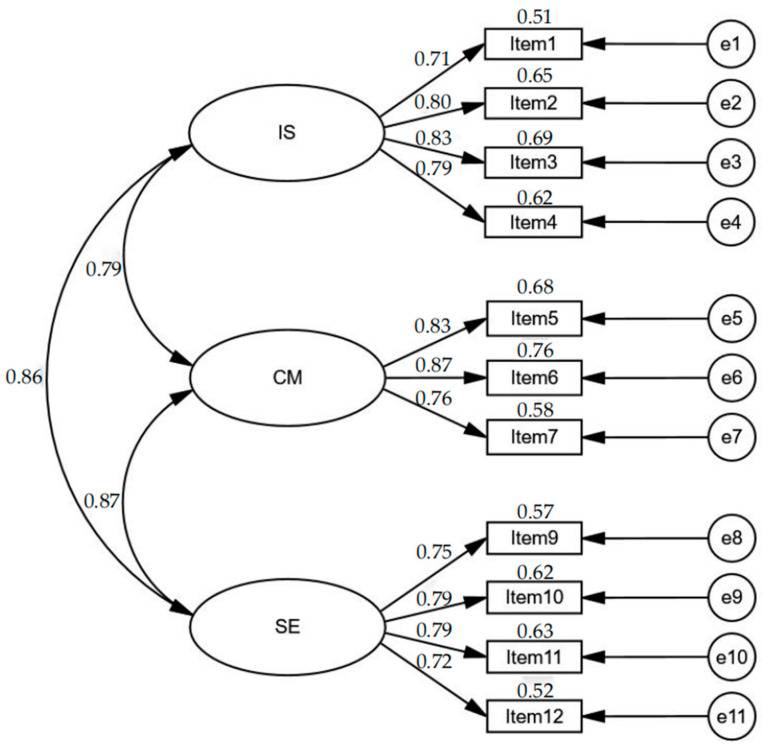
The best-fit model of TSES-SF (Model 4). Note. IS: “Efficacy for instructional strategies”; CM: “Efficacy for classroom management”; SE: “Efficacy for student engagement”.

The outcomes of the overall model fit, as Table 4 indicates, showed that Model 4 displayed the best fit compared to the other five models. The IFIs were sufficient for all six models (ranging from 0.91 to 0.96 > 0.90). In Model 1 and Model 2, none of the indices except IFI were sufficient. In Model 3 and Model 5, both the RMSEA and TLI (NNFI) were not satisfied (Model 3: RMSEA = 0.085 > 0.08, TLI = 0.94 < 0.95; Model 5: RMSEA = 0.088 > 0.08, TLI = 0.94 < 0.95). Both Model 4 and Model 6 showed a relatively good fit according to the overall model fit indices. However, the differences in AIC and ECVI values led us to prefer Model 4. Figure 1 displays the factor structure of Model 4.

#### 3.2.3. The Internal Structure Model Fit

Regarding the internal structure model fit, it was discovered that the individual item reliability of all observations in Model 3, Model 4, and Model 6 was adequate (Model 3: 0.50 to 0.71 > 0.50, Model 4: 0.51 to 0.76 > 0.50, Model 6: 0.53 to 2.07 > 0.50). In contrast, some observations in Model 1, Model 2, and Model 5 were not satisfied (Model 1: 0.41 to 0.62, Model 2: 0.43 to 0.67, Model 5: 0.48 to 0.76). As Table 5 illustrates, the CR of the three latent variables in Model 3, Model 4, and Model 5 all met the criterion of greater than 0.70 (Fornell & Larcker, 1981). The AVE of the three latent variables in Model 3, Model 4, and Model 5 also satisfied the criterion of greater than 0.50 (Bagozzi & Yi, 1988). However, in Model 1 (CR = 0.94, AVE = 0.56), Model 2, and Model 6, these indices were not met. The analysis revealed that the internal structure fit of Model 3 and Model 4 was good.

To recap, considering that only Model 4 fully met all indices of the preliminary fit, overall model fit, and internal structure model fit, Model 4 was considered the best representation of the C-TSES-SF among EC-PSTs and was employed in the subsequent analysis.

### 3.3. Measurement Invariance

According to the results of the multigroup CFA on Model 4 (C-TSES-SF), as Table 6 summarizes, the ΔCFI and ΔRMSEA values of configural, metric, scalar, and residual invariance across age and college year were all less than or equal to the thresholds of 0.01 and 0.015, respectively (age: ΔCFI ranged from 0.001 to 0.01 ≤ 0.01, ΔRMSEA ranged from 0.001 to 0.003 < 0.015; college year: ΔCFI = 0.002 < 0.01, ΔRMSEA ranged from 0.004 to 0.005 < 0.015). This suggested that configural, metric, scalar, and residual invariance were established across age and college year.

### 3.4. Concurrent Validity and Convergent Validity

Table 7 shows that in the C-TSES-SF sub-scales, SE had very significant extremely high-level correlations with IS and CM, respectively, with *r* ranging from 0.73 to 0.76 (below 0.80); IS had a large level correlation with CM, with an *r* of 0.69 (below 0.80). This finding supports the concurrent validity among the three C-TSES-SF sub-scales. Furthermore, the results in Table 7 demonstrate that the convergent validity of the C-TSES-SF was satisfactory, with significant extremely high-level correlations (*p* < 0.001) between the C-TSES-SF and its three sub-scales (IS: *r* = 0.90, CM: *r* = 0.89, SE: *r* = 0.92).

### 3.5. Criterion-Related Validity

Regarding the criterion-related validity of the C-TSES-SF, the results (Table 8) indicated that all factors from the C-TSES-SF were significantly positively correlated with the STPIS and its sub-scales. Specifically, the STPIS, PE, and PW exhibited a significant large correlation strength with the C-TSES-SF, IS, CM, and SE, respectively. PVa showed a significant large correlation strength with the C-TSES-SF and CM, and a medium correlation strength with the IS and SE. PVo displayed a significant large correlation strength with the C-TSES-SF, CM, and SE, and a significant medium correlation strength with the IS. These findings support the criterion-related validity of the C-TSES-SF.

### 3.6. Reliability

The C-TSES-SF’s Cronbach’s alpha coefficient was 0.93, and its sub-scales internal consistency scores were IS (*α* = 0.86), CM (*α* = 0.86), and SE (*α* = 0.85), respectively. In addition, the *ρ*-values were 0.92 for the C-TSES-SF, 0.81 for IS, 0.81 for CM, and 0.80 for SE individually. These results indicated that the C-TSES-SF’s internal consistency reliability and composite reliability were excellent, and the internal consistency reliability and composite reliability of its three sub-scales were good.

## 4. Discussion

This study addressed a significant gap in the literature by adapting the C-TSES-SF and examining its factor structure, validity, measurement invariance, and reliability in the contexts of Chinese EC-PSTs. Our findings suggest that the C-TSES-SF is best represented by a modified three-factor model for EC-PSTs, which offers a more culturally relevant and context-specific framework for assessing TSE. The psychometric properties of the C-TSES-SF were robust, confirming its reliability and validity in measuring TSE among Chinese EC-PSTs across different ages and college years. This study enhances the understanding of TSE among Chinese EC-PSTs and contributes to more accurate assessments of TSE in the Chinese ECE context by incorporating cultural and educational context-specific adjustments.

While previous studies have considered various rival models for the TSES-SF, including single-factor, two-factor, three-factor, and complex bi-factor models (Tschannen-Moran & Hoy, 2001; Tsui & Kennedy, 2009; Ruan et al., 2015; Lu et al., 2021; Chan et al., 2024), we evaluated the construct validity of the C-TSES-SF using more stringent criteria (preliminary fit, overall fit, and internal structure fit). This approach may be more advantageous as it provides detailed information regarding the model’s structure. Results indicate that TSE of Chinese EC-PSTs is best represented by a modified three-factor model, excluding item 8, including efficacy for instructional strategies (IS), efficacy for classroom management (CM), and efficacy for student engagement (SE). Our findings confirm that TSE is a multi-dimensional, stable construct, as described by W. K. Hoy and Woolfolk (1990). It also provides empirical evidence that the TSES-SF is a successful instrument for measuring the nature of TSE. In essence, the three-factor solution identified in this study effectively captures the two dimensions of TSE (as stated earlier) as defined in the integrated model proposed by Tschannen-Moran et al. (1998). Specifically, first of all, the three-factor model (IS, CM, and SE) reflects “the analysis of the teaching task” dimension, which assesses “what will be required of them in the anticipated teaching situation” (Tschannen-Moran et al., 1998, p. 231) (i.e., instruction, management, and engagement). Secondly, the “self-perception of teaching competence” dimension reflects how teachers assess their current abilities in relation to the tasks required in teaching and is measured by all the questions in the TSES-SF, which typically focus on teachers’ current competence and their confidence in their teaching effectiveness. For example, “To what extent can you use a variety of assessment strategies”?

In addition, our empirical data also suggest that the three dimensions of efficacy (IS, CM, and SE) are critical to understanding and developing Chinese EC-PSTs’ TSE. In the context of Chinese early childhood education (ECE), shaped by Confucian cultural values, ISTs, and PSTs are required not only to manage classrooms (Tsui & Kennedy, 2009) but also to foster young children’s sense of collectivism (Ministry of Education of the People’s Republic of China, 2001) and support their socioemotional development (Stevenson et al., 1993). Therefore, classroom management and student engagement play a crucial role in EC-PSTs’ TSE. Instructional strategies are also critical because ISTs and PSTs must adapt to various play-based teaching methodologies, especially in environments where ECE is rapidly evolving. Meanwhile, the three dimensions of efficacy can provide effective guidance for teacher training and development. Through the use of the TSES-SF, teacher education programs can target EC-PSTs development in the core areas of instructional strategies, classroom management, and student engagement.

Moreover, our results suggest that the TSE factor structure (concept) among Chinese EC-PSTs is also multi-dimensional, as in the case of general ISTs in previous studies (Tschannen-Moran & Hoy, 2001; Ruan et al., 2015; V. T. Ho et al., 2023). Specifically, our study confirms the initial three dimensions (IS, CM, and SE) of TSE proposed by Tschannen-Moran and Hoy (2001) based on American ISTs. Given the cultural differences between America and China, our findings support the generality and cross-cultural validity of the basic theoretical construct for TSE. In other words, teachers (including PSTs) across diverse educational systems face similar key challenges in evaluating their professional competence within these core tasks of teaching despite cultural differences.

The poor fit of item 8 (“How well can you establish a classroom management system with each group of children”) may be attributed to the influence of Confucian culture, which is characterized by collectivism (Sun et al., 2019). Classroom instruction in the Chinese education system emphasizes discipline, values conformity, whole-group teaching, and collective learning (Smith & Hu, 2013; B. Y. Hu et al., 2017). Further, the competitive national admission test in Chinese society, shaped by Confucian values, leaves a lack of space for collaboration and communication among students (children), and limits opportunities for questioning or discussion during teachers’ instruction (Holmes, 2005; Thanh, 2012). Consequently, grouping children for differentiated instruction is uncommon in China (Ruan et al., 2015). This contrasts with the individualistic culture of American society (Sun et al., 2019), where diverse forms of instruction such as group discussions, inquiries, and collaborations are more prevalent in school instruction (Smith & Hu, 2013), as reflected in item 8 of the original TSES-SF. Our findings align with previous studies on ISTs in four Asian Confucian culture countries (Ruan et al., 2015; Sun et al., 2019; V. T. Ho et al., 2023), suggesting that the structure of TSES-SF is stable across Confucian cultural contexts and highlighting the influence of Confucian cultural values on TSE. Additionally, PSTs’ educational perspectives on ECE may also have an impact on the poor fit of item 8. Given EC-ISTs and EC-PSTs have different functions and responsibilities than teachers at other stages of education, ECE places greater emphasis on developing children’s appropriate habits and attitudes (Lin & Gorrell, 2001). On the one hand, in the context of ECE in Asia, socioemotional development is widely recognized as a necessary prerequisite for the development of intelligence (Stevenson et al., 1993). One of the socioemotional goals of ECE in China is to cultivate young children’s sense of collectivism and affection for their hometown and nation (Ministry of Education of the People’s Republic of China, 2001). On the other hand, EC-ISTs and EC-PSTs are supposed to play a key role in the social development of young children, assisting them in adjusting to the social structure and rules of their future schools (Sun et al., 2019). Therefore, the Chinese EC-PSTs are not familiar with the “classroom management system with each group” as mentioned in item 8, which is also evident from the fact that the mean score for item 8 among Chinese EC-PSTs is the lowest (*M* = 5.65) among the 12 items. The above reasons may explain why Chinese EC-PSTs have different potential perspectives on TSE. This finding is noteworthy as it highlights that cultural values, social and educational contexts, as well as professional backgrounds may influence the appropriateness of the TSES-SF (Tsui & Kennedy, 2009; Dilekli & Tezci, 2020), especially in PSTs (Lin & Gorrell, 2001).

We then assessed the measurement invariance of the C-TSES-SF across age and college year using multigroup CFA. Although age and college year groups are usually strongly correlated, they reflect overlapping but distinct aspects of EC-PSTs’ experiences. Age reflects maturity and life-stage differences, whereas college year reflects the progression through a structured teacher training program. In many contexts, age and college year may not align perfectly for EC-PSTs in China due to distinctions in primary school entry age, differences in educational systems, and factors such as retaking the college entrance examination, delayed enrollment, or academic suspension. Therefore, testing measurement invariance across these groups remains necessary to ensure the broader applicability of our findings, even in cases where age and college year do not correlate strongly. Compared to previous studies, our findings revealed robust measurement invariance of the TSES-SF across age and college year, for the first time, indicating that there are no differences in the C-TSES-SF’s three-factor model (Model 4) across various ages and college years among Chinese EC-PSTs. Given the measurement invariance is a prerequisite of the comparison of group means (Putnick & Bornstein, 2016), our findings are meaningful as they provide evidence for comparisons of TSE across age and college year for EC-PSTs in China. Moreover, our outcomes also provide the feasibility of evaluating the effectiveness of relevant targeted intervention measures.

Noticeably, we excluded the measurement invariance testing of the C-TSES-SF across genders from the present study. This decision was due to the direct influence of sample size on the *χ*^2^ statistic and the precision of factor loading parameter estimation (Meade, 2005). Furthermore, in small samples (*n* < 100), absolute fit indices such as RMSEA tend to over-reject well-fitting models (Chen et al., 2008; Putnick & Bornstein, 2016). Given the limited number of male participants (*n* = 31), conducting such an analysis would likely yield unstable and unreliable results.

The convergent validity and criterion-related validity of C-TSES-SF, as well as concurrent validity among its three sub-scales, were evaluated using Pearson correlation analyses. Our results demonstrated sufficient convergent and concurrent validity. Interestingly, the correlation coefficients between the three sub-scales of the C-TSES-SF in this study, with *r* between 0.69 and 0.76, were higher than those reported in a Vietnamese ISTs study (*r* ranging from 0.31 to 0.52) with the same factor structure (V. T. Ho et al., 2023). This may reflect the stage of professional development of EC-PSTs, who are in the early stages of their teaching careers and lack extensive teaching experience. Thus, unlike ISTs, EC-PSTs may view IS, CM, and SE as interrelated aspects of teaching rather than distinctly separate dimensions. Worth mentioning, as such data were not reported in previous studies with the same factor structure (Ruan et al., 2015; Burgueño et al., 2019), future research could explore these relationships in diverse teacher populations to assess their generalizability across professional development stages. Additionally, our findings present evidence for the criterion-related validity of the C-TSES-SF by validating the correlation between the STPIS and the C-TSES-SF. Compared to previous studies (Poulou, 2007; Nie et al., 2012; Burgueño et al., 2019; Lu et al., 2021; Salas-Rodríguez et al., 2021; V. T. Ho et al., 2023), our results enhance the understanding of TSES’s criterion validity in EC-PSTs. This is achieved by introducing a new questionnaire to verify the TSES’s criterion-related validity, building on earlier research that demonstrated a correlation between self-efficacy and PI (J. Zhang, 2019; Hong et al., 2021). This finding aligns with prior research suggesting that PI is an important factor in assessing the validity of TSE measures, particularly among EC-PSTs (Marschall, 2022; Zarrinabadi et al., 2023; Matsuda & Hamada, 2024).

The *α* and *ρ* values provide evidence for the good reliability of the scale. The *ρ* values were consistent with those obtained in a prior study among PSTs in Spain (Burgueño et al., 2019). However, the *α* values in this study were higher than those in previous studies among ISTs (Tschannen-Moran & Hoy, 2001; Salas-Rodríguez et al., 2021; V. T. Ho et al., 2023), indicating superior internal consistency in this study and contributing new evidence to knowledge about the internal consistency of the TSES-SF among ISTs and PSTs. Our findings indicate that the TSES-SF is applicable in different cultural and teacher contexts.

It is worth mentioning that we compared TSE differences between age group 1 (18–20 years, *n* = 229) and age group 2 (21–24 years, *n* = 173), as well as between college year group 1 (freshmen and sophomores, *n* = 213) and college year group 2 (juniors and seniors, *n* = 189). Due to the small number of male EC-PSTs (31 out of 402), as mentioned earlier, we did not perform a gender-based comparison. The results showed that EC-PSTs in the 21–24 age group exhibited slightly higher TSE than those in the 18–20 age group (*M_age_*_18–20_ = 5.86 vs. *M_age_*_21–24_ = 5.89). Similarly, EC-PSTs in the juniors and seniors group demonstrated higher TSE than those in the freshmen and sophomores group (*M_freshmen & sophomores_* = 5.84 vs. *M_juniors & seniors_* = 5.91). These findings suggest that teaching experience may have a greater impact on EC-PSTs’ TSE than age or maturity. This aligns with a previous study (Lin & Gorrell, 2001), which found that TSE is influenced by teaching experience.

Based on these findings, teacher education programs may consider increasing the number of teaching practicums for EC-PSTs to more effectively promote TSE development. Furthermore, future research could focus on longitudinal studies that track TSE development over time, particularly as EC-PSTs transition from their teacher education programs to becoming full-time teachers. This would provide valuable insights into the factors influencing TSE development throughout the early stages of their teaching careers.

Finally, our EC-PST participants demonstrated lower TSE compared to EC-ISTs (*M_PSTs_* = 5.87 vs. *M_ISTs_* = 6.83) in Hong Kong (Leung et al., 2023), potentially due to the more practical experience of ISTs positively affecting their TSE. This explanation is speculative, and it is important to note that a comparison of specific factors between ISTs and PSTs cannot be realized due to the use of a two-factor structure in the TSES-SF adopted for ISTs. Therefore, future research should further collect qualitative data from EC-ISTs to better understand the reasons behind this difference.

### Limitations

This study has several limitations. Firstly, the generalizability of our findings to other regions in China may be limited as the sample was drawn from a single university in Hainan province. Future research should consider samples covering a wider range and different educational levels to improve the generalizability of the study findings. Secondly, we did not assess the psychometric properties of the long-form TSES, which could be considered in future research to facilitate further cross-cultural comparisons among EC-PSTs using different forms of the TSES. Thirdly, a limitation of this study is the inability to conduct measurement invariance analysis across gender due to the small number of male participants (*n* = 31). Future studies should prioritize recruiting more gender-balanced samples to facilitate robust and reliable measurement invariance analysis. Lastly, another limitation is the small number of senior participants (*n* = 18, representing the fourth year). This may be due to the increased academic and career pressures faced by seniors during their final semester, including completing their graduation thesis, attending job interviews, and preparing for further studies, which may limit their availability for campus-based activities such as this study. Future research should explore ways to increase senior participation as they are about to enter the teaching profession and possess more time and experience to develop their TSE.

## 5. Conclusions and Contribution

This study fills a gap in understanding the TSE of EC-PSTs in Chinese ECE contexts by providing robust empirical evidence on the factor structure, validity, measurement invariance, and reliability of the C-TSES-SF. Importantly, our findings enhance the cross-cultural applicability of the TSES, demonstrating its appropriateness in Chinese-speaking Confucian cultural contexts.

Additionally, our findings have key practical implications. First, the C-TSES-SF provides a reliable tool for assessing TSE in EC-PSTs, which is essential for designing targeted interventions among teacher training programs to improve teaching effectiveness. As part of an ongoing quasi-experimental study on the effects of instructional strategies on EC-PSTs’ TSE in Hainan, as mentioned earlier, this research offers an evidence-based foundation for enhancing their TSE. Second, our research results can also contribute to education policy making (such as ECE quality assessment standards) and curriculum design (such as the teacher training content framework) to help ensure the overall quality of future early childhood teachers and better meet the needs of China’s rapidly developing ECE environment.

## Figures and Tables

**Table 1:** Sociodemographic descriptions of the sample.

Variables	N (%)	SD	Variables	N (%)	SD
Gender		0.27	Home Province		8.32
Male	31 (7.7)		Hainan	144 (35.8)	
			Anhui	10 (2.5)	
Female	371 (92.3)		Fujian	14 (3.5)	
Age		1.35	Gansu	6 (1.5)	
18	22 (5.5)		Guangdong	10 (2.5)	
			Guangxi	20 (5.0)	
19	75 (18.7)		Guizhou	18 (4.5)	
			Hebei	9 (2.2)	
20	132 (32.8)		Henan	21 (5.2)	
			Heilongjiang	1 (0.2)	
21	100 (24.9)		Hubei	7 (1.7)	
			Hunan	21 (5.2)	
22	40 (10)		Jilin	6 (1.5)	
			Jiangxi	10 (2.5)	
23	21 (5.2)		Liaoning	7 (1.7)	
			Inner Mongolia	5 (1.2)	
24	12 (3.0)		Qinghai	4 (1.0)	
College year		0.87	Shandong	5 (1.2)	
Freshman	94 (23.4)		Shanxi	6 (1.5)	
(first year)			Shaanxi	9 (2.2)	
Sophomore	119 (29.6)		Sichuan	17 (4.2)	
(second year)			Tibet	16 (4.0)	
Junior	171 (42.5)		Xinjiang	14 (3.5)	
(third year)			Yunnan	7 (1.7)	
Senior	18 (4.5)		Zhejiang	7 (1.7)	
(fourth year)			Chongqing	8 (2.0)	

Note: Percentages may not sum to exactly 100% due to rounding.

**Table 2:** Dimensions of the TSES-SF.

Factor	Item	Item No.
IS	1. To what extent can you use a variety of assessment strategies?	4
	2. To what extent can you provide an alternative explanation or example when children are confused?	
	3. To what extent can you craft good questions for your children?	
	4. How well can you implement alternative strategies in your classroom?	
CM	5. How much can you do to control disruptive behavior in the classroom?	4
	6. How much can you do to get children to follow classroom rules?	
	7. How much can you do to calm a child who is disruptive or noisy?	
	8. How well can you establish a classroom management system with each group of children?	
SE	9. How much can you do to get children to believe they can do well in educational and instructional activities?	4
	10. How much can you do to help your children value learning?	
	11. How much can you do to motivate children who show low interest in educational and instructional activities?	
	12. How much can you assist families in helping their children do well in kindergarten?	

Note. IS: “Efficacy for instructional strategies”; CM: “Efficacy for classroom management”; SE: “Efficacy for student engagement”.

**Table 3:** Descriptive statistics for the TSES-SF.

Item	Sample (*n* = 402)					
	M	SD	Skewness	Std. Error	Kurtosis	Std. Error
1	5.72	1.22	−0.03	0.12	0.44	0.24
2	6.06	1.19	0.00	0.12	0.20	0.24
3	6.00	1.26	−0.33	0.12	0.48	0.24
4	5.72	1.26	0.02	0.12	0.09	0.24
5	5.67	1.23	−0.11	0.12	−0.33	0.24
6	5.88	1.24	−0.06	0.12	−0.31	0.24
7	5.82	1.23	0.13	0.12	0.04	0.24
8	5.65	1.24	−0.07	0.12	−0.12	0.24
9	6.14	1.20	0.06	0.12	−0.26	0.24
10	6.08	1.16	−0.11	0.12	0.03	0.24
11	5.86	1.16	−0.07	0.12	−0.06	0.24
12	5.66	1.28	0.01	0.12	−0.31	0.24

**Table 4:** Overall model fit for existing models.

	No. of Factors	Item No.	*χ* ^2^	df	*χ*^2^/df	GFI	RMSEA	NFI	IFI	TLI (NNFI)	CFI	AIC	ECVI
Model 1	1	12	337.53	54	6.25	0.87	0.114	0.89	0.91	0.89	0.91	385.53	0.96
Model 2	2	12	314.00	53	5.92	0.88	0.111	0.90	0.92	0.90	0.92	364.00	0.91
Model 3	3	12	197.24	51	3.87	0.92	0.085	0.94	0.95	0.94	0.95	251.24	0.63
Model 4	3	11	142.67	41	3.48	0.94	0.079	0.95	0.96	0.95	0.96	192.67	0.48
Model 5	3	11	168.74	41	4.12	0.92	0.088	0.94	0.95	0.94	0.95	218.74	0.55
Model 6	Bi-factor model	12	134.44	44	3.06	0.95	0.072	0.96	0.94	0.96	0.97	202.44	0.51

Note. Model 1 and Model 3 were established by Tschannen-Moran and Hoy (2001); Model 2 was recommended by Tsui and Kennedy (2009); Model 4 was proposed by Ruan et al. (2015); Model 5 was suggested by Lu et al. (2021); Model 6 was provided by Chan et al. (2024), with the residual variance of item 1 set to 0; *χ*^2^: “Chi-square”; df: “degrees of freedom”; *χ*^2^/df: “Chi-square by degrees of freedom ratio”; GFI: “goodness of fit index”; RMSEA: “root mean square error of approximation”; NFI: “normed fit index”; IFI: “incremental fit index”; TLI (NNFI): “Tucker–Lewis coefficient (Bentler-Bonett non-normed fit index)”; CFI: “comparative fit index”; AIC: “Akaike information criterion”; ECVI: “expected cross validation index”.

**Table 5:** AVE and CR for existing models.

	CR				AVE			
	IS	CM	SE	TS	IS	CM	SE	TS
Model 2	-	0.89	-	0.89	-	0.62	-	0.54
Model 3	0.86	0.88	0.85	-	0.61	0.64	0.58	-
Model 4	0.86	0.86	0.85	-	0.61	0.67	0.58	-
Model 5	0.87	0.86	0.83	-	0.58	0.67	0.61	-
Model 6	0.36	0.31	0.57	-	0.19	0.13	0.51	-

Note. CR: “composite reliability”; AVE: “average variance extracted”; IS: “Efficacy for instructional strategies”; CM: “Efficacy for classroom management”; SE: “Efficacy for student engagement”; TS: “Teaching and Support”.

**Table 6:** Measurement invariance across age and college year.

Model	*χ* ^2^	df	CFI	RMSEA	Δ*χ*^2^	Δdf	ΔCFI	ΔRMSEA
Age								
Configural invariance	235.38	82	0.945	0.068	-	-	-	-
Metric invariance	241.94	90	0.946	0.065	6.56	8	0.001	0.003
Scalar invariance	256.99	101	0.944	0.062	15.05	11	0.002	0.003
Residual invariance	301.46	118	0.934	0.062	44.47 ***	17	0.010	0.001
College Year								
Configural invariance	244.81	82	0.941	0.070	-	-	-	-
Metric invariance	247.32	90	0.943	0.066	2.51	8	0.002	0.004
Scalar invariance	251.79	101	0.945	0.061	6.98	11	0.002	0.005
Residual invariance	264.32	118	0.947	0.056	12.53	17	0.002	0.005

Note. Model comparison: *** *p* < 0.001; *χ*^2^: “Chi-square”; df: “degrees of freedom”; CFI: “comparative fit index”; RMSEA: “root mean square error of approximation”; Δ*χ*^2^: “Chi-square change”; Δdf: “degrees of freedom change”; ΔCFI: “comparative fit index change”; ΔRMSEA: “root mean square error of approximation change”.

**Table 7:** Correlations between the C-TSES-SF and sub-scales.

	C-TSES-SF	IS	CM	SE
C-TSES-SF	1			
IS	0.90 ***	1		
CM	0.89 ***	0.69 ***	1	
SE	0.92 ***	0.73 ***	0.76 ***	1

Note. *** *p* ≤ 0.001; C-TSES-SF: “Chinese Version of Teachers’ Sense of Efficacy Scale Short Form”; IS: “Efficacy for instructional strategies”; CM: “Efficacy for classroom management”; SE: “Efficacy for student engagement”.

**Table 8:** Correlations between the C-TSES-SF and STPIS.

	C-TSES-SF	IS	CM	SE
STPIS	0.56 ***	0.45 ***	0.62 ***	0.48 ***
PVa	0.30 ***	0.23 ***	0.34 ***	0.25 ***
PE	0.55 ***	0.45 ***	0.63 ***	0.44 ***
PW	0.39 ***	0.34 ***	0.39 ***	0.33 ***
PVo	0.33 ***	0.23 ***	0.36 ***	0.32 ***

Note. *** *p* ≤ 0.001; C-TSES-SF: “Chinese Version of Teachers’ Sense of Efficacy Scale Short Form”; IS: “Efficacy for instructional strategies”; CM: “Efficacy for classroom management”; SE: “Efficacy for student engagement”; STPIS: “Student Teacher Professional Identity Scale”; PVa: “Professional values”; PE: “Professional efficacy”; PW: “Professional willingness”; PVo: “Professional volition”.

## Data Availability

The raw data supporting the conclusions of this article will be made available by the corresponding author on request.

## Notes

1 The sampled university was selected because it has the longest history (from 1956) of offering ECE programs in Hainan, with the largest number of EC-PSTs enrolled, making it a representative site for this study. Additionally, approximately 80% of EC-ISTs in Hainan are graduates of this university.

2 Such as Curriculum Standards of Teacher Education and Professional Competency Standards of Normal Students of Preschool Education.

3 Such as Certification Standards for Pre-school Education Major.

4 Such as Implementation Plan for Undergraduate Education Evaluation in Higher Education Institutions (2021–2025).

## Abbreviations

The following abbreviations are used in this manuscript:

TSE	Teachers’ sense of efficacy
TSES	Teachers’ Sense of Efficacy Scale
TSES-SF	Teachers’ Sense of Efficacy Scale Short Form
C-TSES-SF	Chinese Version of Teachers’ Sense of Efficacy Scale Short Form
IS	Efficacy for instructional strategies
CM	Efficacy for classroom management
SE	Efficacy for student engagement
TS	Teaching and Support
PI	Professional identity
STPIS	Student Teacher Professional Identity Scale
PW	Professional willingness
PVa	Professional values
PE	Professional efficacy
PVo	Professional volition
PSTs	Pre-service teachers
EC-PSTs	Early childhood pre-service teachers
ISTs	In-service teachers
EC-ISTs	Early childhood in-service teachers
ECE	Early childhood education
SD	Standard deviation
M	Mean
EFA	Exploratory factor analysis
CFA	Confirmatory factor analysis
KMO	Kaiser-Meyer-Olkin
*χ*^2^/df	Chi-square by degrees of freedom ratio
CFI	Comparative fit index
TLI (NNFI)	Tucker–Lewis coefficient (Bentler-Bonett non-normed fit index)
RMSEA	Root mean square error of approximation
GFI	Goodness of fit index
NFI	Normed fit index
IFI	Incremental fit index
AIC	Akaike information criterion
ECVI	Expected cross validation index
AVE	Average variance extracted
CR	Composite reliability

## Appendix A

### Appendix A.1

**Table A1:** Chinese version of the TSES-SF.

TSES-SF Chinese Version								
1	2	3	4	5	6	7	8	9
完全不能	微乎其微	很小程度	略有一些	一定程度	相当多	比较大程度	接近非常大	非常大程度
教学策略效能感								
1. 你能在多大程度上使用不同的教育评价方法?								
2. 当幼儿感到困惑时，你能在多大程度上为其提供另一种解释或例子呢?								
3. 你能在多大程度上为幼儿创设一个好的问题?								
4. 你能在课堂上很好地实施替代策略吗?								
班级管理效能感								
5. 你能在多大程度上控制课堂上幼儿破坏课堂秩序的行为?								
6. 你能在多大程度上让幼儿遵守课堂的规则?								
7. 你能在多大程度上让一个吵闹或说话的幼儿安静下来?								
8. 你多大程度上能为班级里不同小组的幼儿建立课堂管理体系?								
幼儿参与效能感								
9. 你能多大程度上让幼儿树立自己能在教育教学活动中有良好表现的信心?								
10. 你能在多大程度上帮助幼儿重视学习?								
11. 你能在多大程度上激励那些对教育教学活动兴趣低下的幼儿?								
12. 你能在多大程度上指导家长，使其协助他们的幼儿在幼儿园有良好的发展?								

### Appendix A.2

**Table A2:** Original version of the TSES-SF.

Original TSES-SF (Tschannen-Moran & Hoy, 2001)								
1	2	3	4	5	6	7	8	9
Nothing	Negligible	Very Little	A slight Degree	Some Degree	A Fair Amount	Quite a Bit	Almost a Great Deal	A Great Deal
Efficacy for instructional strategies (IS)								
1. To what extent can you use a variety of assessment strategies?								
2. To what extent can you provide an alternative explanation or example when students are confused?								
3. To what extent can you craft good questions for your students?								
4. How well can you implement alternative strategies in your classroom?								
Efficacy for classroom management (CM)								
5. How much can you do to control disruptive behavior in the classroom?								
6. How much can you do to get students to follow classroom rules?								
7. How much can you do to calm a student who is disruptive or noisy?								
8. How well can you establish a classroom management system with each group of students?								
Efficacy for student engagement (SE)								
9. How much can you do to get students to believe they can do well in schoolwork?								
10. How much can you do to help your students value learning?								
11. How much can you do to motivate students who show low interest in schoolwork?								
12. How much can you assist families in helping their children do well in school?								
